# Virus-free transfection, transient expression, and purification of human cardiac myosin in mammalian muscle cells for biochemical and biophysical assays

**DOI:** 10.1038/s41598-023-30576-1

**Published:** 2023-03-12

**Authors:** Lok Priya Velayuthan, Luisa Moretto, Sven Tågerud, Marko Ušaj, Alf Månsson

**Affiliations:** Department of Chemistry and Biomedical Sciences, Linnaeus University, 391 82 Kalmar, Sweden

**Keywords:** Biochemistry, Biological techniques, Biophysics, Cell biology, Molecular biology

## Abstract

Myosin expression and purification is important for mechanistic insights into normal function and mutation induced changes. The latter is particularly important for striated muscle myosin II where mutations cause several debilitating diseases. However, the heavy chain of this myosin is challenging to express and the standard protocol, using C2C12 cells, relies on viral infection. This is time and work intensive and associated with infrastructural demands and biological hazards, limiting widespread use and hampering fast generation of a wide range of mutations. We here develop a virus-free method to overcome these challenges. We use this system to transfect C2C12 cells with the motor domain of the human cardiac myosin heavy chain. After optimizing cell transfection, cultivation and harvesting conditions, we functionally characterized the expressed protein, co-purified with murine essential and regulatory light chains. The gliding velocity (1.5–1.7 µm/s; 25 °C) in the in vitro motility assay as well as maximum actin activated catalytic activity (k_cat_; 8–9 s^−1^) and actin concentration for half maximal activity (K_ATPase_; 70–80 µM) were similar to those found previously using virus based infection. The results should allow new types of studies, e.g., screening of a wide range of mutations to be selected for further characterization.

## Introduction

Myosins are molecular motors which develop force and motion by interacting with actin filaments in a cyclic process driven by ATP turnover. This process is the basis for a variety of important functions, such as muscle contraction, non-muscle cell motility/force-development, intracellular cargo transport and cell signaling^1^. Up to 79 classes^2^ were recently identified in the myosin superfamily. All of them are built around a myosin heavy chain (MHC; ~ 90–250 kD) with actin-binding site, catalytic ATPase site and other elements of importance for motor function as well as for formation of filaments and cargo-binding. In addition, light chains, with stabilizing, modulatory and regulatory roles, are attached to each of the heavy chains. For insights into basic mechanisms of myosin motor function, but also for detailed studies of disease-causing mutations, it is important to be able to express and purify all the mentioned protein components.

The most used system for expressing and purifying proteins relies on *E. coli* as an expression host resulting in high purification yields (if the expression product is soluble) as well as labor and cost effectiveness^3^. However, the prokaryotic system lacks full cellular machinery to assist appropriate folding and post-translational modification of eucaryotic proteins. Whereas expression and purification of functional myosin light chains is possible using *E. coli*, the myosin heavy chains (MHCs) cannot be produced in functional form using this system^4^. Therefore, a variety of eukaryotic expression and purification systems based on *Dictyostelium*^5–7^, *Drosophila melanogaster*^8^, insect cells^9–14^ and mammalian cells^15,16^ have been developed. Although these systems work well for production of a variety of MHC classes, they have had limited success with vertebrate striated muscle myosins^17–19^, probably due to failure to include all the protein folding machinery of muscle cells. Although a number of papers have identified UNC-45 as a chaperone involved in myosin folding and sarcomere assembly^20–24^, there seem to be other factors involved, such as Hsp70/Hsp90^25^ and chaperonin^26^. In line with this view Winkelmann and colleagues presented evidence that appropriate folding of striated muscle myosins into a fully functional form only occurs in a differentiated muscle-like environment, i.e., muscle myotubes^27–29^. Based on this idea, mouse C2C12 myoblasts that differentiate into myotubes were introduced as an expression host cell line of choice^27^. The myoblasts were transfected with plasmids carrying chicken embryonic skeletal muscle MHC under a promoter, which ensures that MHC expression begins only upon differentiation into myotubes. Purification yield was sufficient to evaluate myosin function based on actin-activated ATPase activity. The generation of stable cell lines^27^ in this early version of C2C12 based expression and purification system (“C2C12 based system” from here) offers relatively high purification yields with time and cost-efficient workflow eliminating transfection cycles. However, the stable cell line development can require up to 12 months^30^ and would not be the best approach for examining several different constructs (e.g. point mutations) of a particular myosin. In contrast, transient expression offers the advantage of short development time and quick turnover^30^. Thus, the C2C12 based system was later modified utilizing transient adenovirus expression to introduce and study the impact of myosin point mutations associated with hypertrophic cardiomyopathies^31^. The same system was further optimized by Resnicow et al.^32^ to allow production of sufficient amounts of MHCs for transient kinetic analysis of recombinant human skeletal and cardiac myosin isoforms^33–35^. Of note, considerably higher ATPase activities were achieved for both wild type (WT) and mutant β-cardiac myosin in these studies than in a previous report^19^ where human β-cardiac myosin mutants were expressed and purified from insect cells. Presently, the adenovirus-based infection of C2C12 myotubes allows production of sufficient amounts of properly folded and functional striated muscle myosins (S1 and HMM-like constructs) for critical functional studies among others, in vitro motility assays and transient biochemical solution kinetics^34,36^.

Whereas the C2C12 based system, relying on viral gene delivery, gives high yield of functional proteins, it is time and work intensive as well as costly compared to other mammalian-cell based systems. This is both due to the gene delivery method based on adenoviruses and the adherent mode of C2C12 cell growth. Regarding the use of adenoviruses, this is time and work demanding e.g., due to requirement of a series of enrichment and purification steps using a human cell line (e.g., HEK293) to obtain sufficient virus titer^37,38^. Other constraints related to virus-based gene delivery include the step-wise cloning of very big plasmids that must include viral genetic sequences^39^. This limits the insert size, and the possibility of mutagenesis of either the virus or the cell line^40^. Furthermore, the handling of viruses poses a potential hazard to personnel and requires higher safety level of laboratory infrastructure with additional training of the staff. These requirements make the C2C12 based system less accessible for routine use^8^. What is lacking in this regard is an effective virus-free transfection method.

Muscle cells, like C2C12 myoblasts, especially in their differentiated form (e.g., myotubes and cardiomyocytes) are, however, known to be hard to transfect^41^. The commonly used nonviral chemical methods for in-vitro gene delivery (e.g. polyethylenimine, calcium phosphate) were frequently associated with low efficiency, cost ineffectiveness and low cell survival^41–47^. Experimental set-ups were thus limited to small scales of low confluency myoblast cultures. Physical methods like gene electrotransfer by electroporation can be highly efficient. However, they are again limited by special equipment requirement and small-scale mode of action^48–50^. For protein purification, it is crucial that the gene delivery method is not only efficient and cost competitive but also readily applicable to larger cell volumes^51^, explaining why transient gene expression in C2C12 cells has been dominated by viral gene delivery^52^.

More recently, however, the availability of nonviral chemical transfection agents has expanded, and the cost has been reduced. Some suppliers even offer protocols (application notes) to transfect C2C12 cells with promised high efficiency. Here we have examined such a commercially available and affordable DNA transfection kit, Polyplus JetPrime. To the best of our knowledge, the transfection reagent JetPrime has not previously been used in the production of striated muscle myosin using C2C12 cells. Its use allowed us to develop a detailed protocol for transient expression and purification of human cardiac myosin heavy chains (β-MHC) in C2C12 cells. We demonstrate the effectiveness of the approach by expressing and purifying human β-MHC followed by validation in functional assays. Overall, the comparison of our results to previous data, relying on virus-based gene delivery^53–55^, show minimal functional differences, provided that the length of the myosin construct and the light chain composition are similar. We foresee that our results should make the C2C12 based expression and purification system more attractive and accessible for striated muscle MHCs production. Not the least, the appreciably faster generation of mutants and safer protein purification than in virus-based systems would be advantageous where the purpose is to study a wide variety of mutations e.g. in (cardio)myopathies^56–58^.

## Results

**Figure 1:**
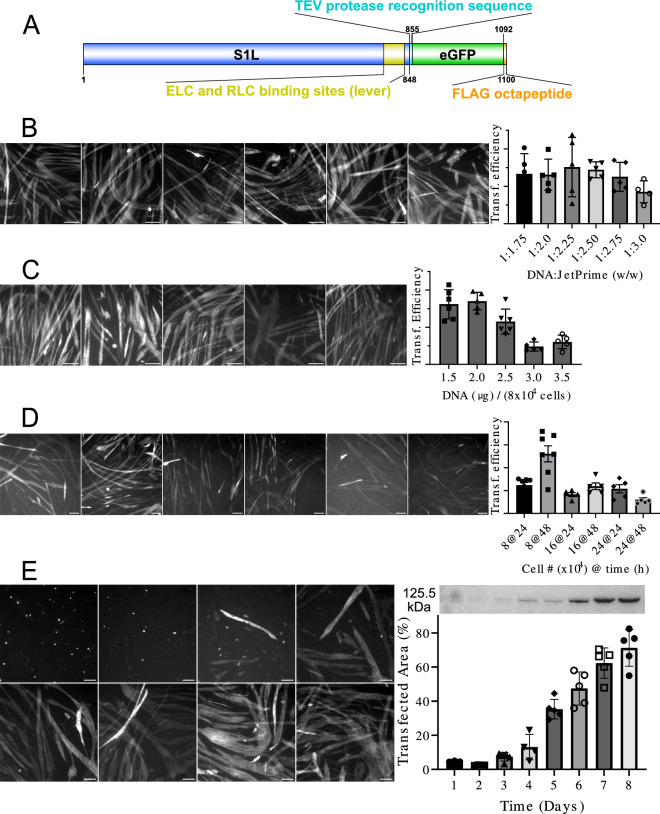
Optimization of transfection and expression. (**A**) Cartoon^64^ of expression construct with denoted amino acid residues of specific parts. (**B**) Transfection efficiency at different DNA:JetPrime ratios. Note relatively broad efficient range where 1:2 was chosen for subsequent experiments. (**C**) Transfection efficiency at different amounts of plasmid (DNA) per seeded cell number (here 8 × 10^4^ cells). The amount of 1.5 µg was chosen for subsequent experiments. (**D**) Transfection efficiency for increasing cell seeding number and two growth periods after seeding (24 and 48 h) before cell transfection. Note that increase in cell number did not improve cell transfection; seeding of the lowest number of cells with growth for 2 days gave best transfection efficiency. (**E**) Transfection efficiency assessed at each of 8 days post transfection. *Right bottom*: Transfection efficiency estimated as fraction of the area with GFP-fluorescence. *Right top:* Western blot using anti-FLAG antibodies used to quantify amount of expressed construct. Original blot is presented in Supplementary Fig. S2. All bar graphs with individual data represent mean ± SD of 3–6 images (field of views) per cell culture dish (well). Scale bars represent 100 µm.

The production of recombinant proteins by means of the transient gene expression technique depends highly on the transfection efficiency. In general, the transfection parameters should be optimized for each combination of transfection reagent and plasmid, carrying the gene of interest (GOI), to ensure efficient protein purification yields. Here we use the transfection reagent JetPrime that, to the best of our knowledge, has not been previously used for production of striated muscle myosin in C2C12 cells. Even though this transfection reagent is accompanied with a protocol (application note) for C2C12 cells, we have re-tested the transfection parameters using our working plasmid, carrying the β-cardiac myosin motor domain construct (S1L; Fig. 1A). This construct, codon optimized for expression in host cell lines of mouse origin, is quite similar to those used previously for virus-based transfection of a long myosin subfragment 1 (aa 1–843; S1L) in C2C12 cells (Table S1). However, our plasmid is fused to enhanced green fluorescent protein (eGFP) with a FLAG tag at its C-terminal, rather than to an Avi-tag as in previous work with S1L. The eGFP is a useful tag in several respects, including for monitoring of the expression progress, surface immobilization in the in vitro motility assay and localization of the myosin motor domain in single molecule studies (cf. preliminary results in Fig. S1). The mode of transfection relies on properly formed DNA:JetPrime complexes. The quantity of both ingredients, and particularly their ratio, are thus important parameters, which can depend on the plasmid used (e.g., due to difference in plasmid size, plasmid structure and conformation). Initially, we therefore tested the transfection efficiency at different DNA:JetPrime ratios. As seen in Fig. 1B, the transfection efficiency stayed optimal for a broad range of ratios, and the efficiency started to decline only at highest ratios tested. Based on the results we have kept the ratio 1:2 for further experiments, as recommended in the company application note. The next parameter we tested was the amount of DNA per seeded number of cells. Company application note suggests 0.75 µg per 4 × 10^4^ cells, seeded into a well of a 24-well plate. For a well of 12-well plate with approximately two times larger well area (as used in our study) this corresponds to 1.5 µg of DNA per 8 × 10^4^ seeded cells per well. We tested the recommended DNA amount and a few higher values, which occasionally can improve low transfection. Results in Fig. 1C demonstrate that higher DNA amounts did not improve transfection efficiency; rather they led to its decline. Thus, we kept the recommended DNA amount per seeded number of cells for further experiments.

As outlined in the Introduction, the proper folding of striated muscle myosins only occurs in differentiated myotubes. Thus, it is important that C2C12 myoblasts enter the differentiation process as soon as possible after transfection. This can most efficiently be achieved when myoblasts are fully confluent. In the manufacturer´s application note, the optimal seeding cell density results at cell confluence of 60–70% at the time of transfection. The lower confluence is usually beneficial for transfection efficiency. However, in our case it delays the most needed myotube differentiation. We thus examined transfection efficiency at several different cell seeding densities and using two different time points for transfection after cell seeding (i.e., cells growing for 24 or 48 h prior to transfection). The aim was to reach near 100% cell confluence at the time of transfection. Our main finding was that use of the recommended cell seeding number of 8 × 10^4^ per well, but transfection 48 h after cell seeding instead of recommended 24 h, resulted in the highest transfection efficiency (Fig. 1D). The cell confluence at that time was also near 100% which assured quick progression to differentiation and myotube formation. Based on these experiments, the recommended cell seeding number was used in our final protocol employing 60 mm diameter culture dishes where the cells were grown for 36 h prior to transfection. Here, 36 h instead of 48 h (Fig. 1D) was used based on pilot experiments with 60 mm cell culture dishes.

Lastly, we determined the optimal cell harvesting time after transfection for highest protein expression. As can be seen from Fig. 1E, the expression increased over time in terms of total transfected cell area as well as the amount of expressed protein, assessed by Western blot, showing a tendency towards saturation at days 7–8.

**Figure 2:**
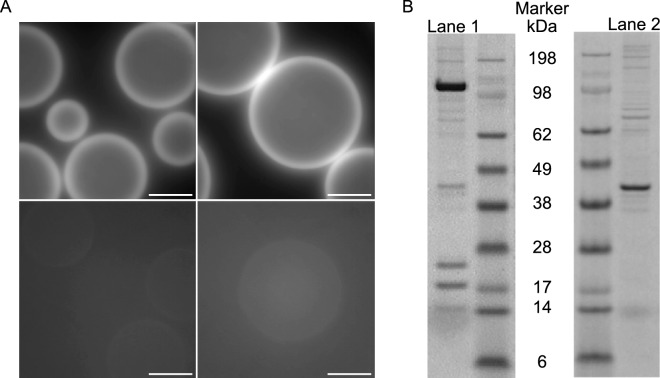
Affinity purification using anti-FLAG antibody resin (beads). (**A**) *Top:* GFP-fluorescence of beads after capturing the expressed proteins via FLAG tag. *Bottom:* Beads (shown under same microscopy conditions as on top) showing lost fluorescence after elution with 3X FLAG peptide. Scale bars: 200 µm. (**B**) *Left:* SDS-PAGE elution analysis showing purified human β-cardiac myosin heavy chain S1L-eGFP-FLAG construct (Lane 1, MHC; 125.5 kDa) co-purified with murine ELCs (~ 23 kDa) and RLCs (~ 19 kDa). *Right:* Mock purification using non-transfected C2C12 cells showing that small protein contaminants (Lane 2) are the results of unspecific cell lysate binding to resin which co-eluted after incubation with 3X FLAG peptide. Original gels are presented in Supplementary Fig. S3.

The above analysis is the basis for the optimized protocol and its adaptation for larger scale transfection using several 60 mm cell culture dishes to express the β-cardiac myosin S1L construct (S1L-eGFP-FLAG, Fig. 1A). This protocol is described in detail in the Experimental Procedures. After harvesting of the cells on day 7 post-transfection, the cells were lysed and the S1L construct was captured from the cell lysate (Fig. 2A top) using a purification resin consisting of micrometer scale beads decorated with anti-FLAG antibodies. The bound protein was eluted by competition with an excess of free FLAG peptide (Fig. 2A bottom). A representative SDS-PAGE protein gel (Fig. 2B) shows purified myosin preparation where the S1L construct is co-purified with endogenous mouse light chains. Some other minor protein contaminants are the consequence of the unspecific cell lysate bindings to anti-FLAG antibodies on purification beads, as suggested by mock purification gel results. As assessed by eGFP absorption at 488 nm up to 0.08 µg and on average 0.04 ± 0.02 µg (mean ± SD, n = 5) per cm^2^ of transfected cells was achieved. Thus, protein purification using cells from ten 60 mm culture dishes yielded up to 13 µg of protein at 1.4 µM concentration.

**Figure 3:**
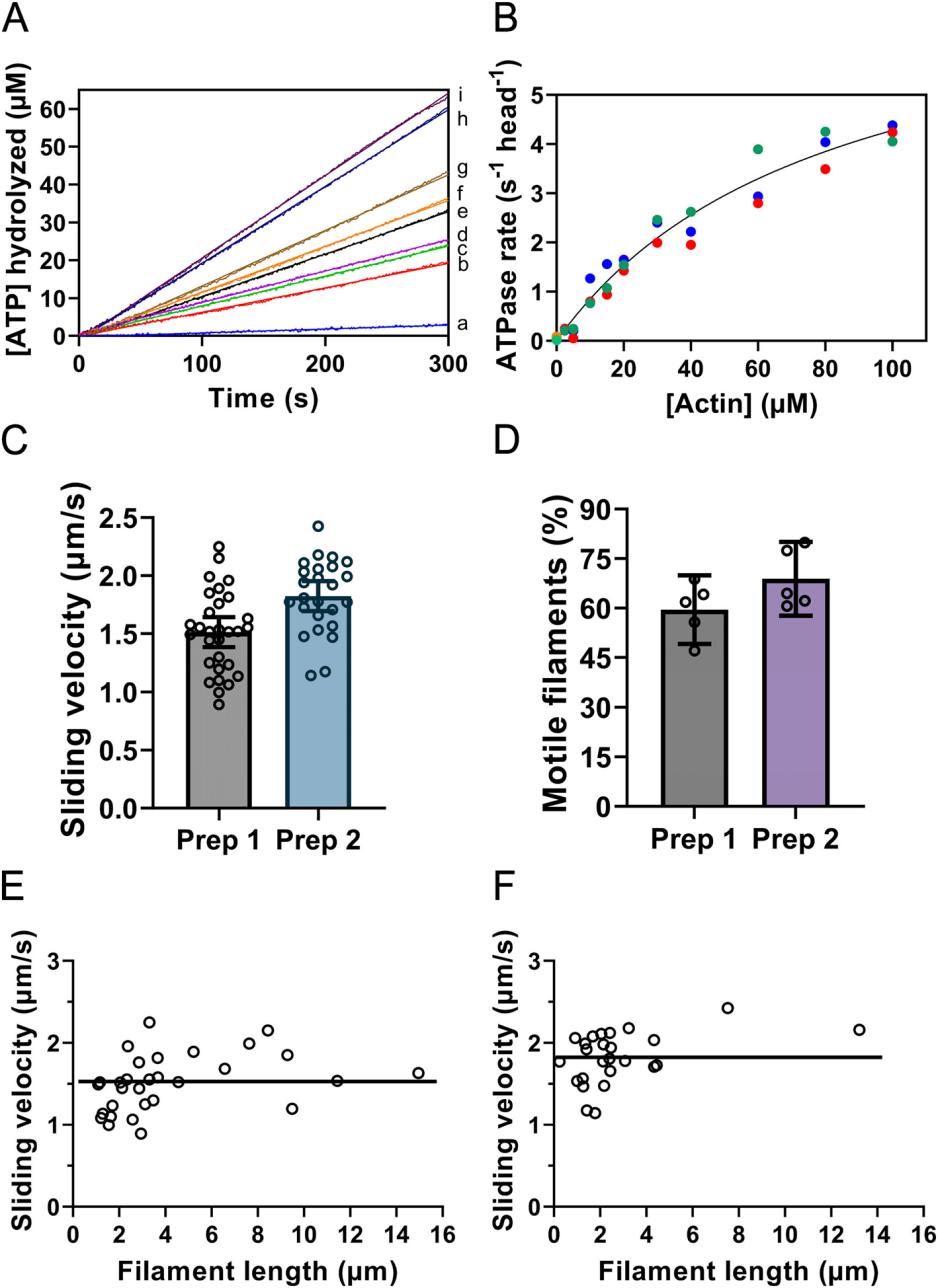
Functional assays of purified human β-cardiac S1L construct co-purified with murine myosin light chains. (**A**) time courses of ATP turnover, using NADH-coupled assay, with constant [S1L-eGFP-FLAG] and [MgATP] but with [F-actin] (concentration relating to monomers) at either 5 (a), 10 (b), 15 (c), 20 (d), 30 (e), 40 (f), 60 (g), 80 (h), or 100 (i). (**B**) Actin-activated steady-state ATPase activity as a function of [F-actin]. The solid line through the data points is the best fit of Eq. (1) to the data with best-fit parameters presented in Table 1. Data from three independent protein preparations shown in different colors. (**C**) The sliding velocities of actin filaments in the in-vitro motility assay at 25 °C for two different protein preparations (Prep 1 and Prep 2). The data are superimposed on mean ± 95% CI from 25–30 filaments for each preparation. (**D**). The fraction of motile filaments after one (for Prep 1) or two (for Prep 2) affinity purifications (“deadheadings”). The data are superimposed on mean ± 95% CI from 5 fields of view per flow cell for each preparation. (**E**) Actin filament sliding velocities versus filament lengths (same data as in C for Prep 1). (**F**) Actin filament sliding velocities versus filament lengths (same data as in C for Prep 2). Straight horizontal lines in E and F indicate mean values suggesting minimal dependence of velocity on filament lengths at lengths > 3 µm.

We then proceeded to characterize the purified myosin construct by measuring the steady-state basal and actin activated ATPase using an NADH-coupled assay. The spectrophotometric data for the steady-state ATPase activity of S1L-eGFP-FLAG is illustrated in Figs. 3A and B with kinetic parameters summarized in Table 1. Actin increased the steady-state ATPase activity of S1L-eGFP-FLAG approximately 200-fold (from *k*_basal_ ~ 0.04 to *k*_cat_ ~ 8 s^−1^), with a *K*_ATPase_ ~ 80 µM, which is the actin concentration at the half maximal ATPase activity (Table 1). A more sensitive assay in terms of the fraction of active heads in the purified S1L-eGFP-FLAG sample, is the in vitro motility assay (IVMA) where actin filaments gliding is observed by surface attached motor proteins. The IVMA results of two independent expression and purification experiments are presented in Fig. 3C and D (Movies S1-S2). Actin filament gliding velocities were on average 1.5 and 1.8 µm/s for the two preparations (Fig. 3C, Table 1). As in previous studies, using a shorter S1 motor fragment (sS1^1-808aa^)^35,59,60^, removal of inactive heads by affinity purification (“deadheading”) was needed to achieve good motility. The fraction of motile filaments (FMF) reached on average 60 and 69%, with one and two deadheadings, respectively (Fig. 3D, Table 1). Unfortunately, we could not find any data for the fraction of motile filaments in previous work using S1L obtained with viral transfection for comparison.

**Table 1:** Summary of ATPase and IVMA results.

Parameters	S1L-eGFP-FLAG
*k*_basal_^§^ (mean ± SEM, n = 3 S1L preparations, 23 °C)	0.04 ± 0.02 s^−1^
*k*_cat_ (mean ± SEM*, n = 3 S1L preparations, 23 °C)	8 ± 1 **s**^−1^
*K*_ATPase_ (mean ± SEM*, n = 3 S1L preparations, 23 °C)	80 ± 20 µM
Actin filament velocity (mean ± SEM, 25 °C, n = 30 filaments, preparation 1)^1^	1513 ± 63 nm/s
Actin filament velocity (mean ± SEM, 25 °C, n = 25 filaments, preparation 2)^2^	1823 ± 63 nm/s
Fraction of motile filaments (mean ± SEM, 25 °Cn = 5 fields of view, preparation 1)^1^	60 ± 4
Fraction of motile filaments (mean ± SEM, 25 °C, n = 5 fields of view, preparation 2)^2^	69 ± 4

^§^measured (NADH-coupled assay).

^*^error of the fitting process of Eq. (1) including data from 3 independent preparations (cf. Fig. 3B).

^1^Single affinity purification (“deadheading”) applied to preparation 1.

^2^Double affinity purification (“deadheading”) applied to preparation 2.

The motor density on the in vitro motility assay surface depends, both on the density of appropriately oriented antibodies and the incubation conditions (time and concentration) for the myosin motor fragments. Whereas we did not measure the motor density, the nearly constant velocity, for actin filament lengths > 3 µm (Fig. 3E, F), suggests that the density is sufficient for attainment of maximum sliding velocity which would then be limited by the cross-bridge detachment rate constant (as in muscle) without effects of the attachment rate^61,62^. A consideration in interpreting the results in Fig. 3E, F is that the velocity is known to saturate for sufficiently long filaments also at low myosin densities but at a lower value^61^. However, we are nevertheless confident that the motor density is sufficiently high in our study to motivate the conclusion that the velocity is detachment limited. First, the saturation occurred already at filament lengths of about 3 µm rather than at longer lengths found at low motor densities in the study of Uyeda et al.^61^. Moreover, as further evidence for a motor density sufficient to give detachment limited velocity, the maximum velocities observed in our study are similar to those found in previous work using adenovirus-based protein production (Table S1 and Fig. S4) and also consistent with those found in muscle cells^63^.

## Discussion

The current state-of-the-art expression and purification system for vertebrate striated muscle myosins relies on viral gene transfer to C2C12 cells. While viral gene transfer ensures high gene delivery efficiency and consequently high protein expression and purification yields, it also has downsides as mentioned in the Introduction (see also Fig. S5). This probably explains its limited implementation worldwide, preventing growth of the research environment, hampering both wide-scale testing of new ideas and broad independent confirmation of previous results. In fact, to the best of our knowledge, only a handful of laboratories world-wide (e.g. reflected in ^32–34,53,65–67^) successfully utilize recombinant vertebrate muscle myosins on a regular basis. We hypothesize that replacing viral gene transfer with a more convenient and user-friendly approach, with respect to both infrastructure and personnel requirement, would be a first step towards broader method usability. We further believe that a first step to achieve this is to demonstrate and fully report a straightforward approach that allows production of purified striated muscle myosin II motor fragments useful in basic functional assays. Our described non-viral transfection method for C2C12 cells based on the transfection reagent JetPrime is such an approach. We first tested and optimized different transfection parameters for transfection of the S1L-eGFP-FLAG construct into C2C12 cells to lay the grounds for the method. In this work we used a construct (see Data availability statement below) that is codon-optimized for expression in the mouse host background. Our tests for optimizing the protocol included varying DNA to JetPrime ratio, [DNA] to cell ratio, cell confluence at time of transfection and finally time of cell harvesting. While some of the tests confirmed parameter values from the company application note, we found that other parameters required further optimization. This is particularly related to the fact that proper muscle myosin folding requires mature muscle conditions which is only ensured in differentiated C2C12 myotubes and not in the myoblast stage. Accordingly, the viral gene transfer is often performed at the myotube stage (however, see^53,66^).

Myotubes or any other differentiated muscle cells are notoriously difficult to transfect using non-viral methods. In the company application note it is therefore instructed to perform JetPrime transfection of myoblasts at 70% monolayer confluence in order to achieve high transfection efficiencies as examined 24 h later. For our purpose, this approach possesses a risk that the expressed myosin will not be properly folded, due to lack of appropriate folding machinery in the myoblasts, leading to accumulation and aggregation of inactive myosin heads. Therefore, important protocol optimizations described here, include (i) transfection of myoblasts at near 100% confluency with their differentiation beginning immediately after the transfection period (4 h) and (ii) carefully selected optimal harvesting day post transfection. Both parameter values were motivated by a report from the Nesmelov group^66^, where, despite using viral gene delivery method, they infected the cells in the myoblast stage at 100% confluence with immediate transition to differentiation medium, followed by harvesting of the myotubes 7 days post infection. Our results show that, on the one hand, the expected high expression efficiency 24 h after transfection (JetPrime C2C12 application note) was sacrificed (Fig. 1E) in favor of faster myotube formation by myoblast fusion. On the other hand, low expression in the first days after transfection works in our favor since only a low fraction of the expressed protein was experiencing non-optimal intracellular environment in terms of appropriate folding machinery. It is of interest to observe how a low transfection area (< 10%) at days immediately post transfection was later expanded and tended to peak at days 7—8 post transfection. This most likely occurred due to horizontal plasmid transfer between few transfected and major non-transfected myoblasts (or transfected with expression below detection limit). Another interesting observation is that this putative process of horizontal gene transfer is accelerated if cells are seeded in a way that near 100% of confluence is reached first within 36–48 h after the seeding, rather than if cells are seeded at a higher density to reach confluence already after 24 h (as is the common way). One may speculate that spontaneously established cell–cell contacts over prolonged growing times before transfection are important for accelerated myoblast fusion, horizontal plasmid transfer and overall transfection efficiency (Fig. 1D). Interestingly, cell harvesting on day 7, post gene introduction was also performed in recently published reports using viral gene transfer to myoblasts^53,66,68^. Longer expression periods can nevertheless be considered in the future. Thus in a recent report with viral transfection the cells were harvested 9–11 days after the myoblast infection resulting in a remarkable purified β-cardiac myosin HMM-like construct yield of up to 262 µg per 150 mm cell culture dish^69^ compared to 33 µg with harvesting after 7 days^53^.

In our protocol, we do not co-transfect, or later exchange, co-purified murine light chains with human cardiac ones as it is generally done in the Spudich/Ruppel^35,36^ and Leinwand labs^33,70^. Instead, the human beta-cardiac MHC is purified together with the murine light chains. This is similar to most other previous protocols relying on virus-based gene delivery^53,65,68,69^. In one study, Swenson et al.^53^ fully identified the murine light chains being found to consist of two essential isoforms (skeletal muscle Myl1, atrial/fetal Myl4) and one regulatory isoform (skeletal muscle Mylpf). In another study where a human β-cardiac myosin HMM-like construct was expressed and purified using C2C12 cells, a third MLC1F isoform was observed^65^. The detection of different light chains partly reflect different lengths of the light chain binding regions in the different MHC constructs but may also suggest differences in C2C12 cellular states between different laboratories. This could be important as cellular state was linked to variability of the myosin ATPase activity measurements obtained within one lab^71^.

Our S1L preparation is fairly pure as indicated in Fig. 2B. However, for further improvements, avoiding minor contaminating proteins, several additional approaches can be adopted in the future. One could thus utilize additional purification by ion exchange chromatography (e.g., HiTrap Q HP column^33,35^) to polish the eluate. Alternatively, one may engineer a TEV restriction site between the eGFP and the FLAG tag to allow cutting the bound constructs from the purification beads^35^. Using the latter approach, the elution by competitive binding of FLAG peptide would not be necessary. Avoidance of this step would most likely eliminate contamination with nonspecific proteins from the cell lysate that might bind to the FLAG-resin purification beads and be released upon elution. Finally, a superior purification tag can be used (e.g., HaloTag^72^). However, that has not been attempted here as we have aimed to avoid changes in the established purification protocol beyond the gene delivery method that is in primary focus of our study.

The yield of our described virus-free C2C12 based expression and purification system is up to 0.08 µg of homogenous protein from every cm^2^ of cultured cells. This is about 2–4 times lower than reported yields of viral C2C12 systems using similar cardiac myosin construct^53–55^ and 4 to 8 times lower than reported for other striated muscle myosin isoforms of similar lengths and using an alternative tag (His-tag)^34^. The yield is also 8–21 times lower (or 6–17 times in mol) than reported recently for a cardiac heavy meromyosin (HMM) like construct^69^. The lower yield with non-viral transfection is expected because currently there is no equally effective alternative to the gene delivery machinery of viruses. However, importantly, the yield of active protein, using our virus free method, is sufficient for steady-state ATPase and in vitro motility assays.

Clearly, the yield of our small scale expression system is too low to allow full characterization of the myosin motor function using transient kinetics or structural studies using e.g. cryo-EM. Scaling up to allow such studies would make the method prohibitively expensive due to the cost of the transfection reagent itself. However, for small scale S1L production, as described here, the method is more cost and time effective, as well as more generally accessible, than the adenovirus based method. This suggests a strategy, e.g. for the study of a wide range of mutations, with 1. initial functional screening (using ATPase and in vitro motility assays) of a wide range of mutations produced on a small scale using the present method, possibly followed by 2. selection of the most interesting mutations for upscaling of the protein production to allow transient biochemical kinetics and structural studies. The latter upscaling could be achieved, once a limited number of mutations have been selected, either using adenovirus based transient expression or another rather lengthy process, namely generation of stable cell lines^27^. The latter may be useful if substantial amounts of a given construct are of interest to produce repeatedly. Such stable C2C12 cell lines can, after appropriate enrichment, express large amounts of protein. Because of lower sensitivity to limited transfection efficiency non-viral liposome based (lipofectin) transfection agent was used in the generation of the stable cell lines^27^.

In addition to the strategies considered above, we also foresee developments using single molecule assays to substitute many of the transient biochemical kinetics analyses. Such developments, if they will be possible to realize, would allow an almost full biochemical characterization using proteins produced by the present non-viral transient expression method. In addition, one may also in the future, increase the yield as well as reducing the cost of non-viral transfection. Thus, to boost the expression further, certain adaptations of the described protocol can be made. Currently, the cell differentiation and protein expression are done in a standard differentiation medium which includes 2% horse serum^32,34,35^. However richer differentiation/expression media formulations have been implemented; most recently a differentiation medium including 10% horse serum and 1% fetal bovine serum has been successfully used^53,69^. Such formulation can potentially boost the protein expression also in our system. Indeed, it has been found that minimal amounts (e.g., 1%) of bovine serum may be crucial for high protein expression efficiency^73^.

The activity of purified motors was checked by measuring basal and actin activated ATPase at steady-state conditions. Strikingly, the measured steady state parameters i.e., basal activity without actin (*k*_basal_), actin activated ATPase activity (*k*_cat_) and *K*_ATPase_ are in very good agreement with recently reported values (*k*_basal_ ~ 0.02 s^−1^, *k*_cat_ ~ 8–9 s^−1^, *K*_ATPase_ ~ 70–80 µM) using similar (S1L) constructs, co-purified, as here, with mouse light chains but using viral transfection^53–55^ (Table S1; Fig. S4). Furthermore, actin filament sliding velocities obtained in our study (1.5–1.8 µm/s) are also in the same range as observed in the mentioned studies (1.5–1.7 µm/s) (Table S1; Fig. S4).

On the other hand, somewhat different kinetic parameters were found in studies using shorter myosin constructs (sS1^1-808aa^), that harbored only essential (not RLC) but human cardiac light chain^35,59,60,71,74–76^ or the even shorter myosin construct S1^1-787aa^ without light chains^65^. These studies showed lower actin activated ATPase activity parameter values (*k*_cat_ ~ 6 s^−1^, *K*_ATPase_ ~ 40 µM) (Table S2, Fig. S4) except for basal ATPase activity (*k*_basal_ ~ 0.02 s^−1^) which remains similar^77^. Moreover, the actin filament velocities using these shorter constructs were lower (~ 0.9 µm/s) than for S1L (Table S2, Fig. S4). The lower *k*_cat_ values in some of these experiments, using viral transfection, were attributed to variability between C2C12 cellular states^71^ suggesting that it may be prudent to perform experiments in parallel (expressing both wild-type and mutated proteins) when analyzing different disease causing mutation of β-cardiac myosin. Considering the consistently higher *K*_ATPase_ values for S1L with murine light chains than for sS1 with human ELC and consistently higher gliding velocities in the in vitro motility assay, we believe that these effects reflect true differences between the sS1 and S1L constructs rather than methodological peculiarities. In support of this view, the human β-myosin HMM-like construct co-purified with mouse light chains also shows similar velocity of ~ 1.5 µm/s^69^. Different mechanisms behind the observed differences may be considered. As pointed out previously, the different lengths of the light-chain-binding lever arm and the different light chain isoforms are likely to be of importance in these regards^53^. The increased length of the lever arm in S1L compared to sS1 could, in itself, account for the higher gliding velocity^78^. Furthermore, because the gliding velocity produced by S1L with murine light chains, seems to be similar (after temperature correction) as seen in skinned muscle fibers from human ventricles (with all human myosin heavy and light chains)^63^, there seems to be no need to assume additional effects of murine vs human light chains on the gliding velocity.

However, it is important to consider, in this connection, that other studies demonstrate a dramatic effect of essential^79,80^ and regulatory^81,82^ light chain isoforms on both striated muscle myosin ATPase kinetic properties and actin filament gliding velocities. Moreover, when performing experiments in parallel, Tang and coworkers^55^, observed that substitutions of the mouse regulatory chain for the human ventricular regulatory light chain (without exchange of the mouse ELC; study 3.1 vs 3.2 in Table S2) demonstrated reduced *K*_ATPase_ and actin filament velocity by ~ 40% and ~ 15%, respectively (see also Fig. S4). To more definitely address the roles of the different light chains without other complications, all experiments should be performed under similar well defined conditions and with full characterization of the light chain isoforms. Considering some perceived variabilities, as discussed above, such studies seem to be of interest in the future.

## Conclusions

We report a virus-free transfection, transient expression, and protein purification method for production of active human striated muscle myosin in sufficient quantities for biochemical and biophysical analysis. We are confident that our bypassing of the viral production step will contribute to “democratizing” (cf.^83^) the human striated muscle myosin expression and purification system, making it attractive and affordable for a broader scientific community. This, in turn, would be expected to greatly benefit both the speed and quality of scientific discoveries that rely on the use of expressed and purified striated muscle myosin. At this stage, method limitations are lower purification yields in comparison to viral gene delivery as discussed above. Another possible limitation could be the current cost of the JetPrime reagent when conducting large scale cell transfections (~ 15€/60 mm cell culture dish, as estimated from product quote obtained from distributor in Sweden, April 2022). Although fair cost judgment would require estimation of cost for comparable viral titer, it may be still valid to say that the method is the most appropriate for low and middle scale experiments for quick characterization of a wide range of different mutations in parallel, using steady-state and single molecule biochemical and biophysical techniques. When up-scaling is required to other methods, particularly adenovirus based infection is more suitable. Above we propose a strategy to combine our method with adenovirus-based protein production or production using a stable cell line. However, in the future, new transfection reagents suitable for C2C12 cells would most likely become commercially available with possibly better cost effectiveness. Additionally, one can foresee that development of single molecule methods may allow more extensive biochemical characterization requiring only minimal amounts of expressed protein.

## Methods

### Ethical statement

The actin was purified from muscle obtained from a euthanized rabbit, following a procedure approved by the Regional Ethical Committee for Animal Experiments in Linköping, Sweden (ref. 17,088–2020). Immediately before euthanization, the rabbit was anesthetized by an intramuscular injection of 0.25 ml Zoletil with active substances: Zolazepam, 6 mg/kg; Tiletamin, 6 mg/kg and Medetomidin, 0.6 mg/kg. Euthanization then followed, by injection of 2 ml penthobarbital (100 mg/ml) in an ear vein. All procedures were performed by the Linnaeus University veterinarian. The actin was isolated from only one rabbit, motivated by the fact that actin properties were not in focus in the study. Of importance was instead the possible variability between myosin from three different expression-purifications in their interaction with actin (see further below).

### Vectors design and production

The plasmids were designed to contain the human MYH7 gene (https://www.uniprot.org/uniprot/P12883) for human β-MHC and the nucleotide sequence was optimized for expression in mouse muscle C2C12 host background. The full length MYH7 gene was truncated to express, what we denote subfragment 1—long (S1L; amino acids residues 1 to 848 of the human β-MHC), cloned into a pcDNA-3.1 plasmid under the CMV promoter^84^, adding a Tobacco Etch Virus (TEV) protease cleavage site before an enhanced Green Fluorescent Protein (eGFP) tag and a FLAG-tag (GenScript Biotech Corporation). In this paper we refer to this construct as human β-cardiac S1L-eGFP-FLAG or simply S1L. This construct has single motor domain but contains both essential and regulatory light chains binding sites.

### Cell culture and differentiation of C2C12 cells

A vial of the mouse myogenic C2C12 cell line (ATCC CRL 1772) was purchased from Sigma (Sigma-Aldrich, Germany, now Merck). The cell line has been derived by serial passage of primary cultures of adult thigh muscle after injury^85^. For routine culture, C2C12 cells were seeded at a density of 10^3^ cells/cm^2^ (for 3 days growth) or 0.5 × 10^3^ cells/cm^2^ (for 4 days growth). They were then grown in growth medium, GM [DMEM-high glucose no sodium pyruvate (Sartorius, Germany), 10% Fetal Bovine Serum (HyClone), 1% Antibiotic–Antimycotic solution (Gibco) and 2 mM L-glutamine (Sigma)] in polystyrene cell culture flasks or dishes (Sarstedt) at 37 °C in a humidified condition supplied with 5% CO_2_ (Forma Series II 3110 Water-Jacketed CO2 Incubator, Thermo Fisher). The cells were subcultured when the confluency was around 60–70%. Under our cell culture conditions, doubling time was estimated^86^ to be 16 ± 2 h (mean ± SD, n = 43).

For myogenic differentiation, the cells were grown to a confluency of 95% and the medium was replaced with differentiation medium, DM. The latter medium contained DMEM-high glucose (no sodium pyruvate) (Sartorius), 2% donor Horse Serum (HyClone), 1% Antibiotic–Antimycotic solution (Gibco), 2 mM L-glutamine (Sigma), 1% MEM Non-essential amino acid (Gibco), 25 mM HEPES (Gibco) and 1 µM insulin (Sigma-Aldrich, now Merck). The cells were differentiated until day 7 by replacing with fresh DM every 24 h. Only cells between passage numbers 5–15 were used in experiments.

### Non-viral transfection of C2C12 cells

Transient transfection of C2C12 cells was performed by using JetPrime® DNA transfection reagent kit (PolyPlus-transfection® SA, France), denoted “JetPrime reagent” in this paper. In this procedure, we took a starting point in the manufacturer´s application manual for C2C12 cells but optimized the protocol for large scale protein expression.

For transfection efficiency optimization, C2C12 myoblasts were seeded at 8 × 10^4^ cells/well in 12-well plates (Sarstedt) and grown for ~ 24 h to attain a confluence of 70% before transfection, unless stated otherwise. A transfection master mix was prepared by mixing 1.5 μg of plasmid DNA (1 µg/µl) and 3 μl of JetPrime reagent (1:2 ratio) in 100 μL of JetPrime® buffer (proprietary PolyPlus-transfection® SA, France; “JetPrime buffer” below) for each well, unless stated otherwise. The transfection mix was added to the cells dropwise and incubated at 37 °C for 3.5–4 h. The transfection process was stopped by replacing GM with 0.5 ml DM after washing the cells once with 0.5 ml phosphate buffered saline (PBS; all per well). As mentioned previously, the DM was changed every 24 h up to day 6. Transfection and expression efficiency was analysed by fluorescence microscopy. In studies, following S1L expression over time, C2C12 myoblasts were seeded at 30 × 10^4^ cells in individual 35 mm (diameter) culture dishes (Nunc, Thermo Scientific) and grown for ~ 36 h to attain a confluence of 95% before transfection. For each dish, a transfection master mix was prepared by mixing 7.5 μg of plasmid DNA and 15 μl of JetPrime reagent (1:2 ratio) in 200 μL of JetPrime buffer. The transfection mix was added to the cells dropwise and incubated at 37 °C for 3.5–4 h. The transfection process was stopped by replacing GM with 2 ml DM after washing the cells once with 2 ml PBS (all per one dish). As mentioned previously, the DM was changed every 24 h up to day 8. Transfection and expression efficiency were analysed daily (one dish per day) first by fluorescence microscopy, and then, following harvesting of the cells, by Western blot.

For protein purification, C2C12 myoblasts were seeded at 6 × 10^5^ cells/cm^2^ in 60 mm diameter plastic dishes (Sarstedt) and grown for ~ 36 h to attain a confluence of 95% before transfection. A transfection master mix was prepared by mixing 11.25 μg of plasmid DNA (1 µg/µl) and 22.5 μl of JetPrime reagent (1:2 ratio) in 500 μL of JetPrime buffer per each 60 mm dish. The transfection mix was added to the cells dropwise and incubated at 37 °C for 3.5–4 h. The transfection process was stopped by replacing GM with 5 ml DM after washing the cells once with 3 ml PBS. As mentioned previously, the DM was changed every 24 h until day 7. The cells were harvested on day 7. After rinsing with 3 ml cold PBS, the cells were scraped off using a cell scraper with 1 ml cold PBS. The cells were then transferred into a 1.5 ml Eppendorf tube and gently pelleted by centrifugation at 300 g for 5 min at 4 °C. The supernatant was discarded, and the pellet was snap frozen using liquid nitrogen with subsequent storage at − 80 °C.

### Fluorescence microcopy with image processing

Expression of the GFP tagged myosin construct was monitored and analyzed in micrographs obtained by an inverted fluorescence microscope (Axio Observer D1, Zeiss, Germany) equipped with a mercury lamp (HBO 103 W/2, Osram, Germany) and using a 10X objective and FITC filter set. Bright field and fluorescence micrographs were acquired using an EMCCD camera (C9100-12, Hamamatsu photonics, controlled by dedicated HCImage software) with consistent exposure time (150 ms) and gain (100) with 8-bit image depth. Images were acquired in different intervals post transfection up to 8 days. Light intensity was kept as low as possible by a discrete FL attenuator (Cat. # 423,647, Zeiss) kept at position 5 (transmission ~ 20%) and cell exposure to excitation light was minimized to avoid cell phototoxicity. Up to six bright field and fluorescence micrographs were acquired per each parameter or cell culture well/dish from randomly selected locations. To avoid selection bias (unintentional selection of high fluorescence areas) the new location was always selected under bright field illumination comprehensively surveying the entire sample^87^. If needed, image brightness and contrast was adjusted in ImageJ (Image/Adjust/Brightness/Contrast).

Since the cells expressing the myosin construct were always 100% confluent at the time of imaging the transfection efficiency was determined as 1) the fraction of the total fluorescent area acquired in fluorescence images and 2) the fluorescence intensity (mean grayscale level) in those areas. Note, that the latter is only comparable between experiments if image acquisition is done in close periods, preferably the same day, with comparable lamp working hours and the same microscope (acquisition) settings. To facilitate the analysis a macro was written in Imagej (Fiji) with a set of available functions (where “//” indicates comment):

//Background subtraction.run("Subtract Background…", "rolling = 50");

//Image segmentation.setAutoThreshold("Default dark");setAutoThreshold("Default dark no-reset");setThreshold(4, 255);//Threshold values must be adapted based on quality of images.

//measure fraction of the total fluorescent area and mean gray value. In function “Measure” settings it is important to check “limit to threshold” box.run("Measure");

For experiments where images were acquired on the same day, the *Transfection Efficiency* (TE) was calculated as product between fraction of total fluorescent area (A) and florescence intensity (I): TE = A × I (arbitrary units) to facilitate relative comparison between different transfection parameters.

### Western blot analysis

Total protein obtained from cell lysates or from the purified proteins was analyzed using SDS-PAGE and Western blot. For total protein isolates, the cells were lysed on ice for 30 min using RIPA buffer (150 mM NaCl, 5 mM EGTA, 1% Triton X-100, 0.1% SDS, 25 mM Tris–HCl, 1 × Roche protease inhibitor cocktail), under sonication for 30 s (Branson 2510-DTH Ultrasonic Cleaner). This was followed by centrifugation at 17,949 × g (13,000 RPM) using an Eppendorf centrifuge 5430R for 10 min at 4℃. Protein samples were then mixed with Pierce™ LDS non-reducing sample buffer (ThermoFisher) along with 50 mM DTT and heated at 95℃ for 5 min. Electrophoresis was performed on NuPAGE™ 4–12% Bis–Tris gel (Thermofisher) at 90 V for 45 min, later increasing to 140 V for an hour in NuPAGE™ MES SDS running buffer (ThermoFisher). The proteins were transferred to a 0.2 µm PVDF membrane using the Trans-Blot Turbo transfer system (BioRad) at 1 A, 25 V for 30 min. After the transfer, the membrane was incubated in 0.2% blocking buffer (EZ block, Biological Industries) for one hour, washed with TBS-T buffer (20 mM Tris, 150 mM NaCl, 0.1% Tween 20, pH = 7.4–7.6) and then incubated overnight with anti-FLAG antibodies (ab1257, Abcam) at 1:20,000 dilution in blocking buffer. The blot was washed in TBS-T buffer and then incubated with Donkey anti-goat antibodies (ab6885, Abcam) diluted in TBS-T at 1:20,000 for an hour. The membrane was finally washed with TBS-T and the blot was developed using Novex^TM^ECL Chemiluminescent substrate kit (ThermoFisher). Images were obtained using ChemiDoc XRS gel Imaging system (Biorad).

### Protein purification and characterization

The purification strategy (buffer compositions, etc.) was based on a previously described procedure^32,35^. All buffers were degassed prior to their use in the purification. The pellet was resuspended in lysis buffer at pH 7.2 (20 mM imidazole pH 7.2, 100 mM NaCl, 4 mM MgCl_2_, 1 mM EDTA, 1 mM EGTA, 1 mM DTT, 3 mM ATP, 1 mM PMSF, 10% sucrose, 0.5% Tween-20, and 1 × Roche protease inhibitor cocktail) and homogenized using a Dounce homogenizer with 50–70 strokes on ice. The lysed cells were spun down using the MLA-80 fixed angle rotor in an Optima MAX-XP ultracentrifuge at 100,000 × g for 1 h at 4 °C with addition of 1–2 mM fresh MgATP just before the centrifugation. The supernatant was incubated with Anti-Flag resin® M2 affinity gel (Sigma-Aldrich) in an Eppendorf tube for 1.5 h at 4 °C on a nutator, shielding it from light and air by wrapping with aluminum foil and parafilm. In this step we used 20–40 µl of packed resin per 60 mm plate, depending on the fluorescence of transfected cells. After incubation, the beads with Anti-Flag resin were washed twice with 20–25 resin volumes of wash buffer at pH 7.2 (20 mM Imidazole pH 7.2, 150 mM NaCl, 5 mM MgCl_2_, 1 mM EDTA, 1 mM EGTA, 1 mM DTT, 3 mM ATP, 10% Sucrose, 1 × Roche protease inhibitor cocktail) followed by two washes without the addition of ATP and protease inhibitor cocktail. The protein was eluted from the beads using elution buffer at pH 7.2 (20 mM Imidazole pH 7.2, 150 mM NaCl, 5 mM MgCl_2_, 1 mM EDTA, 1 mM EGTA, 1 mM DTT, 10% Sucrose, and 150 μg/ml of 3 × FLAG peptide (Sigma-Aldrich)). The eluate was run over Pierce Micro-Spin columns (Thermo Scientific) to remove residual beads and was then concentrated using Amicon Ultra-0.5 centrifugal filter units. For elutions, low protein binding 1.5 ml microcentrfuge tubes were used (Thermo Scientific). The protein concentration was determined by measuring the absorbance at 488 nm using the eGFP extinction coefficient of 61,000 M^−1^ cm^−1^.

### Actin-activated ATPase assay

All buffers and solutions were degassed prior to the assay. The activity of the expressed β-cardiac myosin construct was determined by NADH-coupled actin-activated ATPase assay at steady state condition^88^. The assay was carried out in ATPase assay buffer (10 mM Imidazole pH 7.5, 5 mM KCl, 1 mM MgCl_2_ and 1 mM DTT) at 23℃ by measuring absorbance at 340 nm using an UV spectrophotometer (UV-1800, Shimadzu) in time-scan mode for 5 min. The apparatus was equipped with a super-micro cell holder to hold a supermicro black cuvette, with working volume range 50–200 µl, effectively reducing the volume of the sample needed. F-actin was purified from rabbit back muscles and was dialyzed thrice in degassed assay buffer^89^. Phalloidin (Merck) was added to actin after dialysis at a 1.1 equimolar concentration to stabilize the filaments. The reaction mixture was directly prepared in the cuvette in assay buffer by diluting myosin to a final concentration of 50 nM with varying actin concentrations ranging from 0 to 100 µM along with 2 mM MgATP and NADH-cocktail solution (1.2 mM NADH, 200 U/ml lactate dehydrogenase, 500 U/ml pyruvate kinase and 2.5 mM phospho(enol) pyruvate). The basal myosin ATPase (*k*_basal_) was measured with myosin at a final concentration of 70–150 nM without actin filaments. The reaction absorbance traces at each actin concentration were plotted as ATP turnover vs time and the slopes (ATPase rates) obtained were normalized to the myosin concentration. The normalized reaction rates plotted against increasing actin concentration followed a rectangular hyperbola and were fitted by the Briggs-Haldane steady-state Eq.^88^

1 $$Rate = v_{0} + \left( {\frac{{k_{cat} \left[ {Actin} \right]}}{{K_{ATPase} + \left[ {Actin} \right]}}} \right),$$

to obtain *k*_cat_ and *K*_ATPase_ as well fitted estimation of *k*_basal_ (*V*_0_) (GraphPad Prism v9)_*.*_ The assays were performed using three individual protein preparations (n = 3).

### In vitro motility assays

The motility of actin filaments produced by the expressed β-cardiac myosin S1L construct was evaluated using a standard in-vitro motility assay procedure^35,90^. Coverslips were coated with 1% nitrocellulose in amyl acetate, air dried, and assembled to form a flow cell as described previously^91^. Initially, the non-functional motor heads were separated from the functional heads by carrying out one to two affinity purifications to improve the fraction of motile filaments^91^. Affinity purification was performed by mixing the stock solution with the expressed protein with actin filaments at a concentration (subunit concentration) 10 times that of the S1L concentration for 5 min on ice followed by addition of 4 mM MgATP and incubation on ice for 5 min. The mixture was then centrifuged at 244,900 × g (75,000 RPM) using a TLA 120.1 rotor in the ultracentrifuge (Optima MAX-XP, Beckman Coulter) for 10 min at 4 °C and the supernatant, containing the functional motors was used for the assay. Low ionic strength solution (LISS, 10 mM MOPS, pH = 7.4 at 25 °C, 1 mM MgCl_2_, 0.1 mM EGTA) was degassed and further supplemented with 50 mM KCl and 1 mM DTT to yield a wash buffer. The flow cell was coated with anti-GFP antibodies (MAB3580, Merck, 6 times diluted in wash buffer) for 2 min and then blocked with 1 mg/ml BSA (2 min, prepared in wash buffer). The affinity purified S1L-eGFP-FLAG protein, diluted in wash buffer (≤ 325 nM; measured before affinity purification), was added to the flow cell with incubation for 5 min. Inactive motors were further blocked by a “blocking actin procedure” as described previously^91^. Non-fluorescent F-actin (500 nM), shredded in wash buffer by pipetting was incubated with 2 mM ATP for 2 min (in wash buffer) followed by wash two times with wash buffer. Finally, actin filaments labelled with rhodamine-phalloidin at a concentration of 15 nM in wash buffer were added and incubated for 2 min followed by a one volume wash. Finally, motility assay solution was added. The assay solution contained LISS (see above), 45 mM KCl, 10 mM DTT, 0.64% methylcellulose, and 2 mM MgATP. It was also supplemented with mixtures for ATP regeneration (200 µg/ml Creatine phosphokinase (CPK), 2.5 mM Creatine phosphate (CP)) and oxygen scavenging (3 mg/ml Glucose, 40 µg/ml catalase, 100 µg/ml glucose oxidase (GOX)). The ionic strength was 60 mM. Movies of actin filament sliding were recorded at 25 ± 1℃ using a Zeiss Axio Observer epifluorescence microscope equipped with a 63X objective (fitted with an objective heater), Cy3 filter set and an EMCCD camera (C9100-12, Hamamatsu photonics, controlled by HCImage software) at 5 frames/s and 16-bit image depth. The sliding velocities were calculated using a custom-made MATLAB program^92^. Fractions of motile filaments were determined using ImageJ^93,94^.

### Statistics and reproducibility

Curve fitting for analysis of actin-activated ATP turnover rate is described above. No statistical hypothesis testing was performed but non-overlapping 95% confidence intervals are assumed to correspond to statistically significant differences. The meaning, and origin, of central measures (arithmetic mean values) and error bars are described in the figure legends and in Tables.

No sample size calculations were performed prior to the experiments as the main purpose was not to demonstrate differences in observed variables between groups or from a given population value. Instead, the goal was to test the level of expression of S1L and the functionality of the purified protein together with information about the variability. The definitions of independent random events (n) are given in the Figure legends and in Table 1.

Statistical analyses, e.g. calculation of confidence intervals and curve fitting were performed using Graph Pad Prism software v. 9.2 (Graph Pad Software LLC).

## Supplementary Information

Supplementary Information 1.

Supplementary Video 1.

Supplementary Video 2.

## Data Availability

Most data generated or analysed during this study are included in this published article (and its Supplementary Information files). Additional data generated during or analysed during the current study are available from the corresponding authors on reasonable request. The DNA sequence encoding human beta cardiac myosin motor domain (S1L) generated during the current study is available in the NCBI GenBank depository (part of International Nucleotide Sequence Collaboration [INSDC]), under accession number OQ092356.
