# Supplementary material for: Virus-free transfection, transient expression, and purification of human cardiac myosin in mammalian muscle cells for biochemical and biophysical assays
Source: Sci Rep. 2023 Mar 12;13:4101. doi: 10.1038/s41598-023-30576-1 (PMC10008826; doi:10.1038/s41598-023-30576-1)
Supplement: Supplementary file 1 — Supplementary Information 1. [file 41598_2023_30576_MOESM1_ESM.docx]

**Virus-free transfection, transient expression, and purification of human cardiac myosin in mammalian muscle cells for biochemical and biophysical assays**

^#^Lok Priya Velayuthan, ^#^Luisa Moretto, Sven Tågerud, Marko Usaj*, Alf Månsson*

**Supporting Tables**

**Table S1.** Steady-state ATP turnover and in vitro motility assay velocity data obtained in previous studies using long subfragment 1 (S1L)

| Study # | 1 | 2 | 3 | 3.1 | 3.2 |
| --- | --- | --- | --- | --- | --- |
| Construct | S1^1-843aa^-Avi tag | S1^1-841aa^-Avi tag | S1^1-841aa^-Avi tag | S1^1-841aa^-Avi tag | sS1^1-841aa^-Avi tag |
| *k*_basal_ (s^-1^) | 0.02 | 0.02 | 0.02 | 0.02 | 0.03 |
| *k*_cat_ (s^-1^) | 8.79 | 8.3 | 8.3 | 5.4 | 5.3 |
| *K*_ATPase_ (µM) | 69.88 | 77.5 | 76 | 53 | 31 |
| IVMA (µm s^-1^) | 1.712 | 1.547 | 1.591 | 1.520 | 1.300 |
| T (°C) | 25-26 | 25-26 | 25-26 | 25-26 | 25-26 |
| Surface | NC-biotin-  streptavidin | NC-biotin-  streptavidin | NC-biotin-  streptavidin | NC-biotin-  streptavidin | NC-biotin-  streptavidin |
| Actin Isoform | Rabbit skeletal | Rabbit skeletal | Rabbit skeletal | Rabbit skeletal | Rabbit skeletal |
| ELC | mouse | Mouse | Mouse | Mouse | mouse |
| RLC | mouse | Mouse | Mouse? | Mouse | human |

*NC: nitrocellulose

**1: ^1^**

**2: ^2^**

**3: ^3^**

ATPase/IVMA buffer: MOPS20 buffer: 10mM MOPS, pH 7.0, 20mM KCl, 1mM EGTA, 1mM MgCl2, 1 mM DTT

| Study # | 1 | 2 | 3 | 4 | 5 | 6 | 7 | 8 | 9 | 10 | 11 |
| --- | --- | --- | --- | --- | --- | --- | --- | --- | --- | --- | --- |
| Construct | sS1^1-808aa^-eGFP | sS1^1-808aa^-  eGFP or  C-tag | sS1^1-808aa^-  eGFP or  C-tag | sS1^1-808aa^-  eGFP or  C-tag | sS1^1-808aa^-  eGFP or  C-tag | sS1^1-808aa^-  eGFP or  C-tag | sS1^1-808aa^-  eGFP or  C-tag | sS1^1-808aa^-  eGFP or C-tag | sS1^1-808aa^-  eGFP or  C-tag | sS1^1-808aa^-  eGFP | sS1^1-787aa^- eGFP |
| *V*_0_ (s^-1^) | <0.2 | <0.2 | n.a. | n.a. | <0.2 | n.a. | n.a. | n.a. | n.a. | 0.022 | <0.2 |
| *k*_cat_ (s^-1^) | 7.4 | 6.0 | 4.5 | 5.29 | 5.7  3.3  5.1 | 5.5 | n.a. | 3.0 | n.a. | n.a. | 5.3, 25°C |
| *K*_ATPase_ (µM) | 38 | 40 | 58 | 50 | 34  23  26 | 53 | n.a. | n.a. | n.a. | n.a. | 22.5, 25°C |
| IVMA (µm s^-1^) | 0.8 | 0.47 (MVEL)  0.78 (Top5%) | 0.86 | 0.96 | 1.1§ (MVEL)  1.3§ (Top5%) | 0.668 (MVES)  0.931 (Top5%) | 0.858 (MVIS)  1.213 (Top5%) | 0.82 | 0.762 | n.a. | 0.8 |
| T (°C) | 23 | 23 | 23 | 23 | 23 | 23 | 23 | 23 | 23 | 23 | 32** |
| Surface | NC+aGFP antibody | NC+SNAP-PDZ18 | NC+SNAP-PDZ18 | NC+SNAP-PDZ18 | NC+SNAP-PDZ18 | NC+SNAP-PDZ18 | NC+SNAP-PDZ18 | NC+SNAP-PDZ18 | NC+SNAP-PDZ18 | n.a. | NC+aGFP antibody |
| Actin Isoform | Chicken skeletal | Chicken skeletal/  Bovine cardiac | Chicken skeletal/  Bovine cardiac | Bovine cardiac | Chicken skeletal/  Bovine cardiac | Rabbit skeletal | Bovine  cardiac | Bovine cardiac | Bovine cardiac | n.a. | n.a. |
| ELC | human | human | human | Human | Human | human | human | Human | Human | human | n.a. |
| RLC | n.a. | n.a. | n.a. | n.a. | n.a. | n.a. | n.a. | n.a. | n.a. | n.a. | n.a. |

**Table S2.** Steady-state ATP turnover and in vitro motility assay velocity data obtained in previous studies using shorter subfragment 1 (S1) with either only human essential light chain (ELC) or without light chains at all

*NC: nitrocellulose, **of the objective

**1: ^4^**

**2: ^5^**

**3: ^6^**

**4: ^7^**

**5: ^8^**

§Estimated from graph, not explicitly given. MVEL, TOP5%

**6: ^9^**

**7: ^10^**

**8: ^11^**

1-8: ATPase buffer: 10 mM Imidazole, pH 7.5, 5 mM KCl, 3 mM MgCl2, 1 mM DTT

1-8: IVMA buffer: 25 mM Imidazole, pH 7.5, 25 mM KCl, 4 mM MgCl2, 1 mM EGTA, 1 mM DTT.

**9: ^12^**

IVMA buffer: 25 mM imidazole, pH 7.5, 25 mM KCl, 4 mM MgCl2, 1 mM EGTA, and 10 mM DTT

**10: ^13^**

Basal ATPase buffer: 25 mM potassium acetate, 10 mM Tris pH 7.5, 4 mM MgCl2, 1 mM EDTA and 1 mM DTT

**11: ^14^**

ATPase buffer: 4mM MOPS, 2mM, MgCl2, pH 7.2.

IVMA buffer: 25mM Imidazole, 25mM KCl, 4mM MgCl2, pH 7.6, 2 mM mercaptoethanol

**
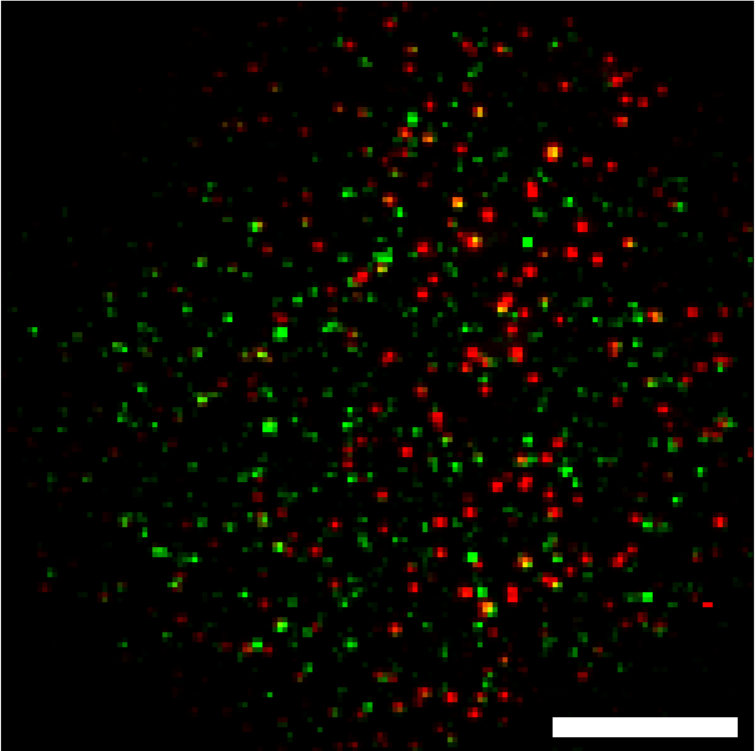
Supporting Figures**

**A**

**B**

**Fig. S1.** Preliminary data of single molecule ATPase using purified eGFP tagged cardiac myosin S1L construct and Alexa647-ATP. A) Merged images of eGFP fluorescence in green and Alexa647-ATP fluorescence in red (time projection of 300 s video, acquired at 20 frames per s) obtained using total internal reflection fluorescence (TIRF) microscopy. Yellow squares represent visible colocalization of both signals. Myosin motors were attached to the surface via GFP monoclonal antibodies deposited on nitrocellulose coated glass slides. Scale bar: 10 µm. B) Representative trace of single molecule basal myosin ATPase depicting characteristic binding events (dwells) from which basal myosin ATPase rates can be estimated. More details regarding methodology can be found in our recent publications ^15-17^


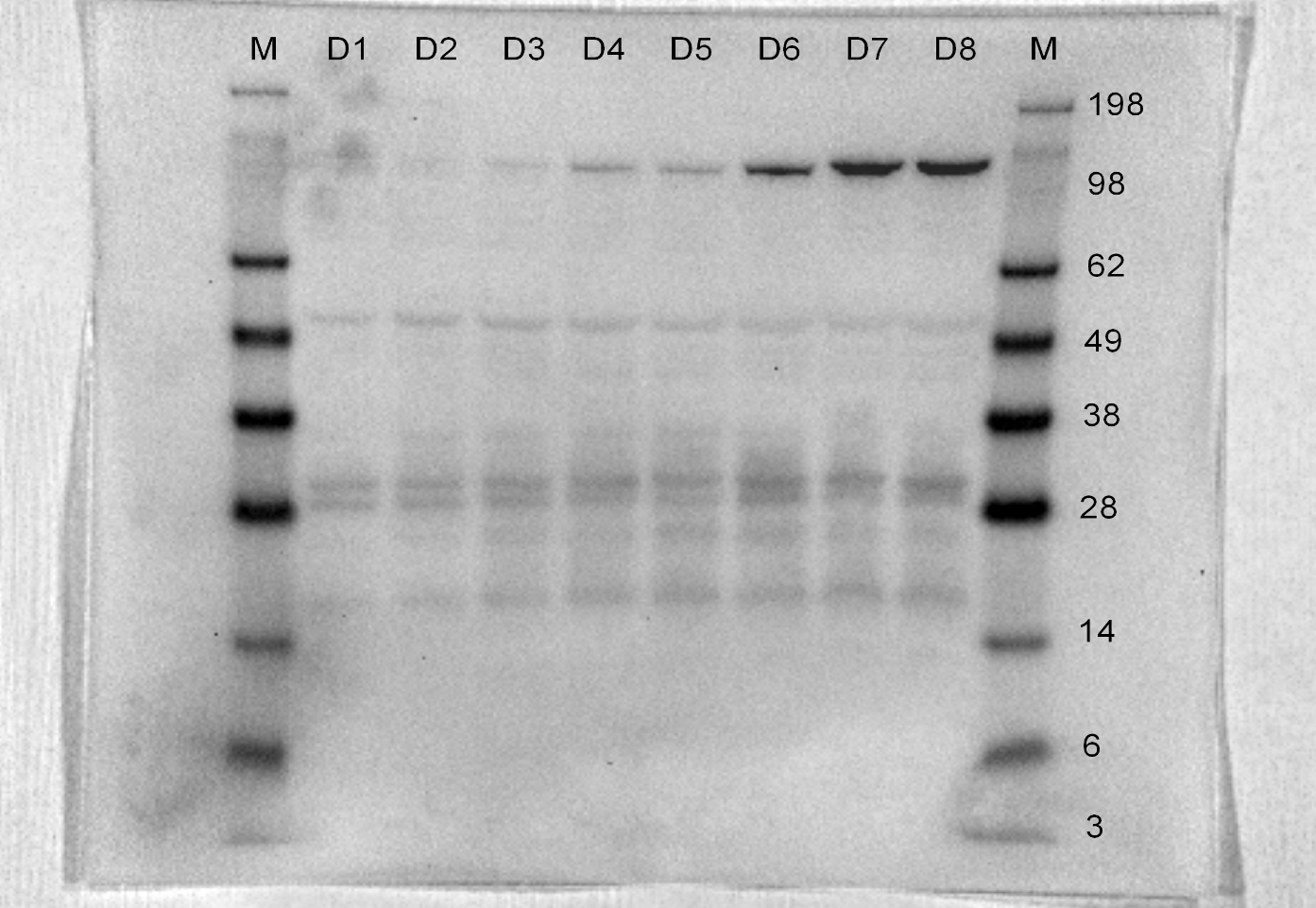


**Fig. S2.** Original membrane of Western Blot using anti-FLAG antibodies for quantification of expressed construct over time (1-8 days (D) post transfection). M – marker with denoted kDa (numbers). Dashed rectangle represents cropped area as presented in Figure 1 (main paper).


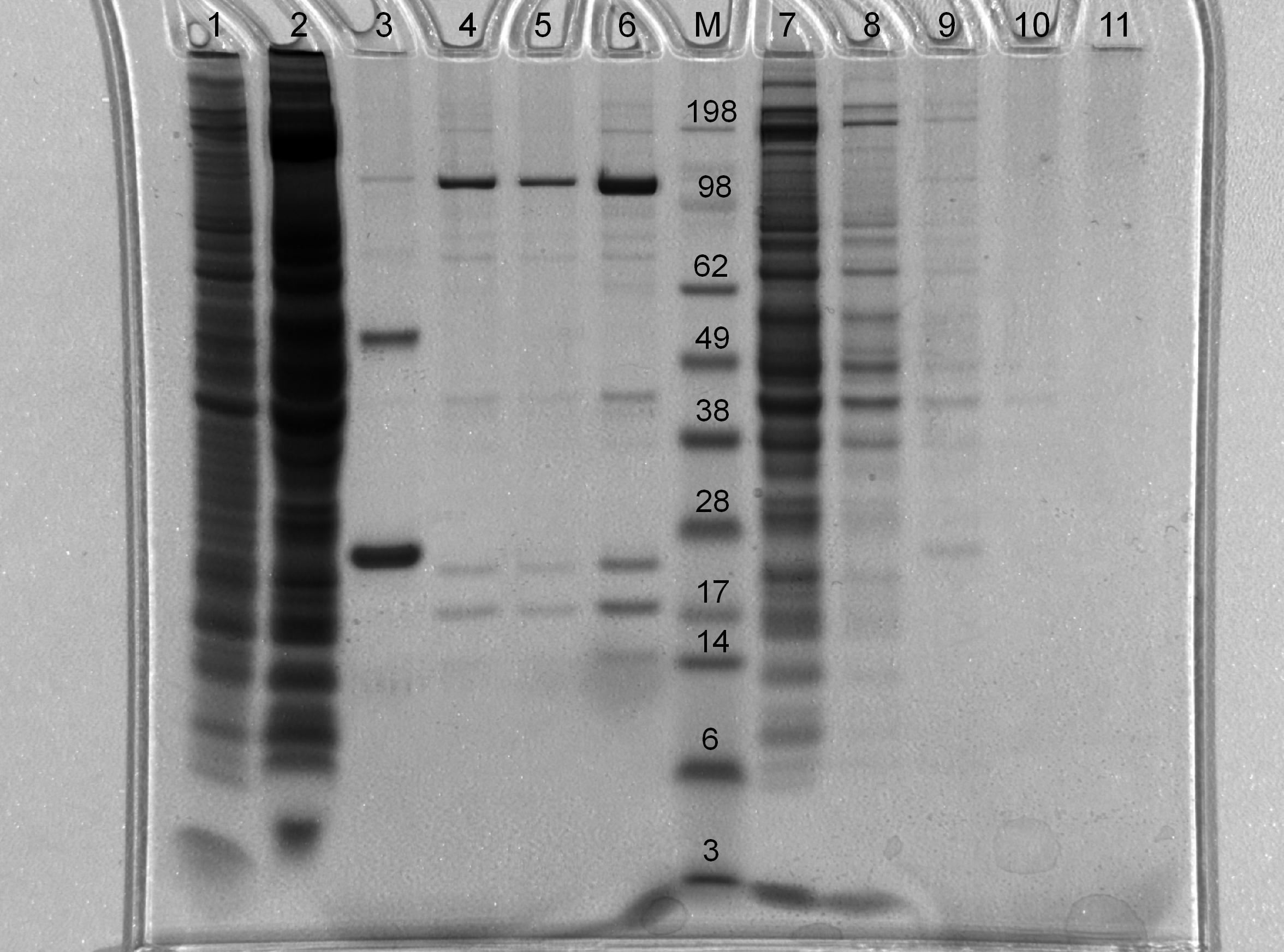


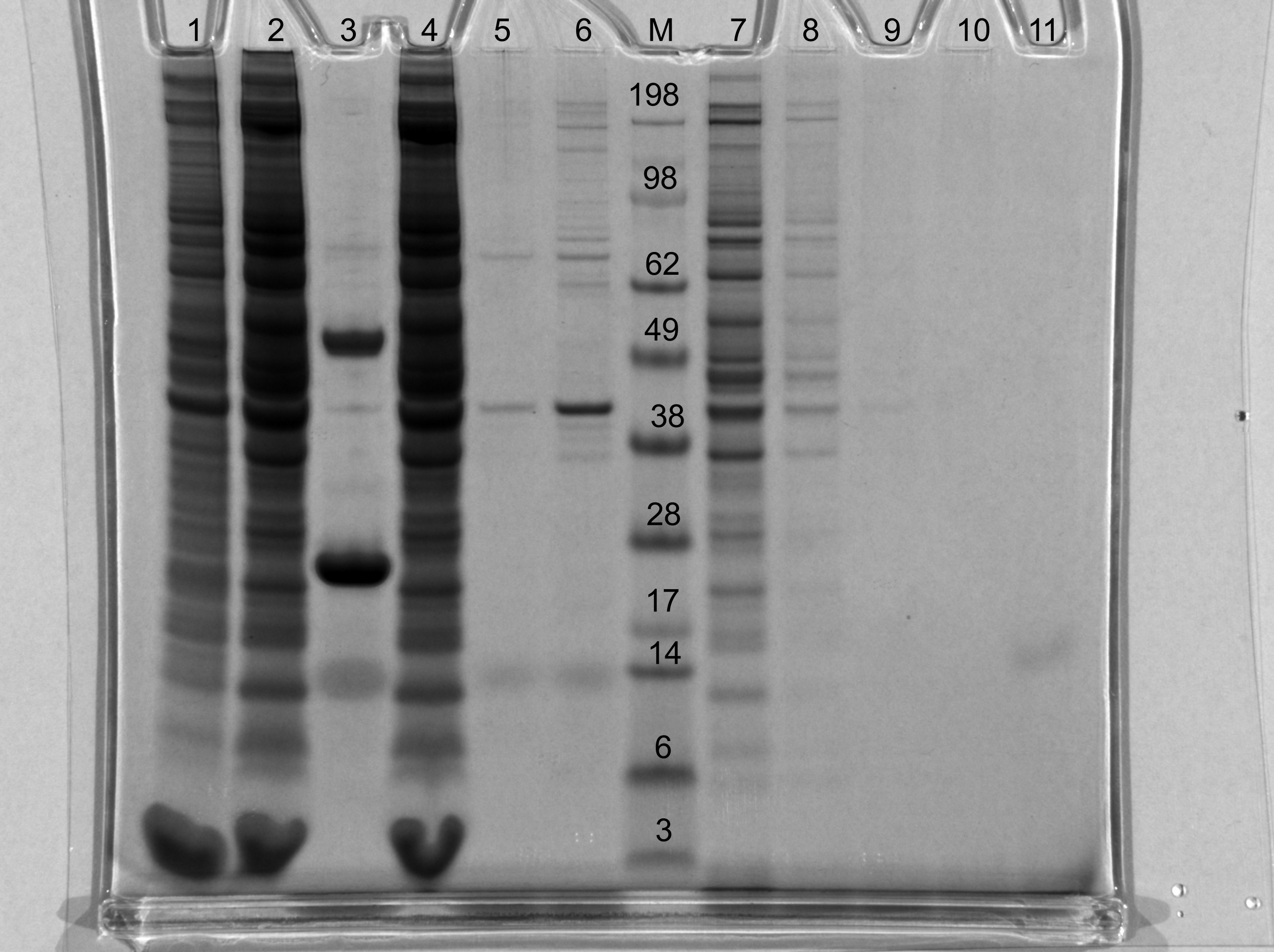


**Fig. S3.** Original SDS-PAGE gels for results presented in main paper Figure 2. *Top:* purification steps for human β-cardiac myosin heavy chain S1L-eGFP-FLAG construct. Lane 1) Pellet, 2) Flow through, 3) Flag-coated beads before elution, 4) Elute-1, 5) Elute-2, 6) Concentrated elute, M-Marker (in kDa), 7) Wash-1, 8) Wash-2, 9) Wash-3, 10) Wash-4, 11) Amicon filtrate. *Bottom:* Mock purification steps. Lane 1) Pellet, 2) Lysate, 3) Beads, 4) Flow through, 5) Elute, 6) Concentrated elute, M-Marker (in kDa), 7) Wash-1, 8) Wash-2, 9) Wash-3, 10) Wash-4, 11) Amicon Filtrate. Dashed rectangles represent cropped areas as presented in Figure 2 (main paper). In the case of the mock purification, the cropped area is also mirrored in the vertical axis in order to have both markers (in kDa) next to each other in Figure 2 to enhance clarity and conciseness of the presentation.

**Fig. S4.** Summary of key data in Tables S1 and S2. Data of our study are encircled. Lines between middle and right bar indicate changes between conditions 3.1 and 3.2 in Table S1.


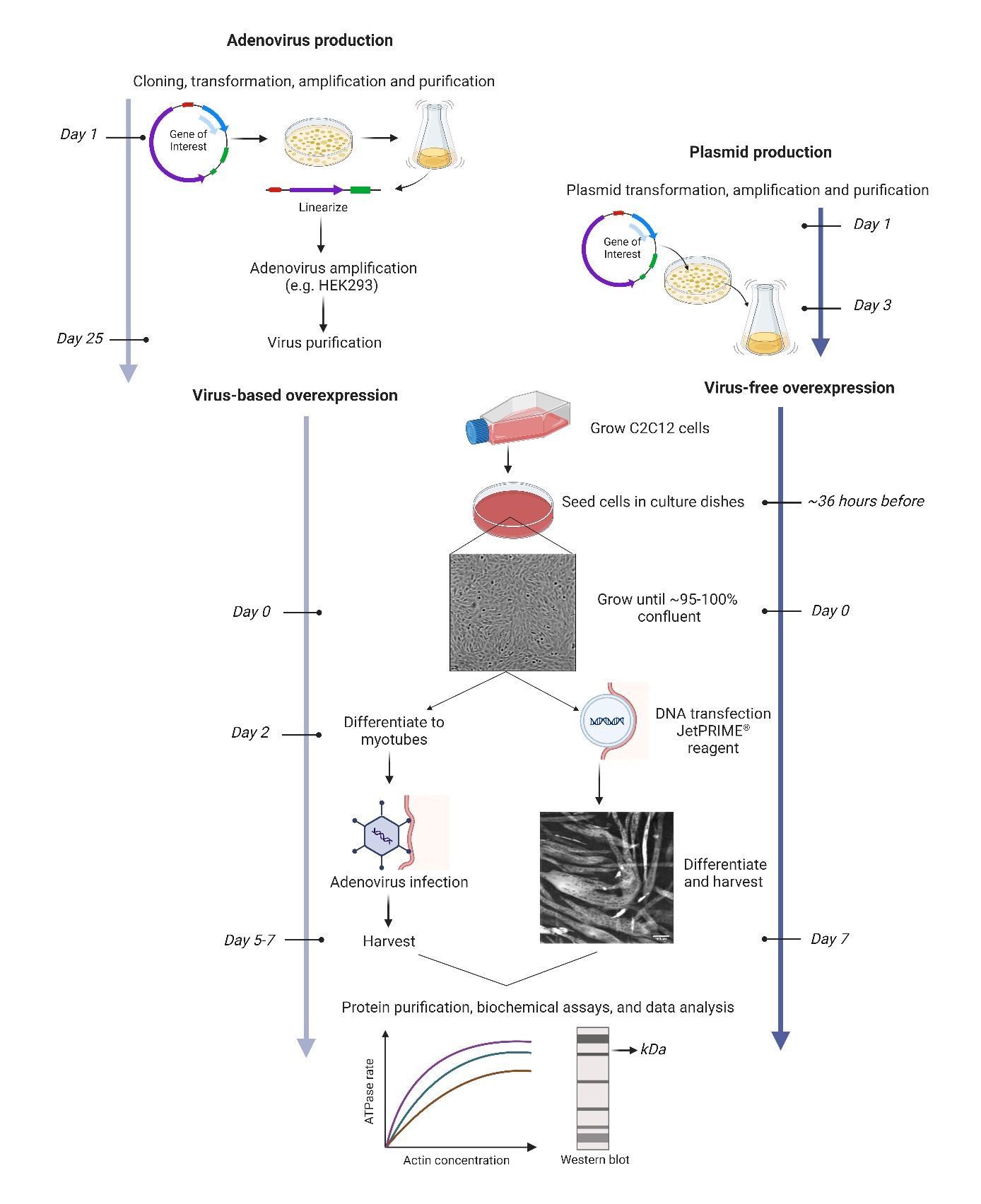


**Fig. S5.** Main differences between protocols for myosin expression in C2C12 cells using virus based and JetPrime based transfection. Figure created with BioRender.com.

**Supporting Movie legends**

**Movie S1**.

Representative in vitro motility assay using purified myosin from Prep 1 (single “deadheading”). Bar represents 10 µm.

**Movie S2**.

Representative in vitro motility assay, using purified myosin from Prep 2 (double “deadheading”). Bar represents 10 µm.

**Supporting References**

1. Swenson, A.M. et al. Omecamtiv Mecarbil Enhances the Duty Ratio of Human beta-Cardiac Myosin Resulting in Increased Calcium Sensitivity and Slowed Force Development in Cardiac Muscle. *J. Biol. Chem.* **292**, 3768-3778 (2017).

2. Tang, W., Unrath, W.C., Desetty, R. & Yengo, C.M. Dilated cardiomyopathy mutation in the converter domain of human cardiac myosin alters motor activity and response to omecamtiv mecarbil. *J Biol Chem* **294**, 17314-17325 (2019).

3. Tang, W., Ge, J., Unrath, W.C., Desetty, R. & Yengo, C.M. Cardiomyopathy mutations impact the actin-activated power stroke of human cardiac myosin. *Biophys J* **120**, 2222-2236 (2021).

4. Sommese, R.F. et al. Molecular consequences of the R453C hypertrophic cardiomyopathy mutation on human beta-cardiac myosin motor function. *Proceedings of the National Academy of Sciences of the United States of America* **110**, 12607-12612 (2013).

5. Nag, S. et al. Contractility parameters of human beta-cardiac myosin with the hypertrophic cardiomyopathy mutation R403Q show loss of motor function. *Science advances* **1**, e1500511 (2015).

6. Adhikari, A.S. et al. Early-Onset Hypertrophic Cardiomyopathy Mutations Significantly Increase the Velocity, Force, and Actin-Activated ATPase Activity of Human β-Cardiac Myosin. *Cell Rep* **17**, 2857-2864 (2016).

7. Aksel, T., Choe Yu, E., Sutton, S., Ruppel, K.M. & Spudich, J.A. Ensemble force changes that result from human cardiac myosin mutations and a small-molecule effector. *Cell Rep* **11**, 910-920 (2015).

8. Kawana, M., Sarkar, S.S., Sutton, S., Ruppel, K.M. & Spudich, J.A. Biophysical properties of human β-cardiac myosin with converter mutations that cause hypertrophic cardiomyopathy. *Science Advances* **3**, e1601959 (2017).

9. Ujfalusi, Z. et al. Dilated cardiomyopathy myosin mutants have reduced force-generating capacity. *Journal of Biological Chemistry* **293**, 9017-9029 (2018).

10. Vera, C.D. et al. Myosin motor domains carrying mutations implicated in early or late onset hypertrophic cardiomyopathy have similar properties. *Journal of Biological Chemistry* **294**, 17451-17462 (2019).

11. Sarkar, S.S. et al. The hypertrophic cardiomyopathy mutations R403Q and R663H increase the number of myosin heads available to interact with actin. *Science Advances* **6** (2020).

12. Vander Roest, A.S. et al. Hypertrophic cardiomyopathy beta-cardiac myosin mutation (P710R) leads to hypercontractility by disrupting super relaxed state. *Proceedings of the National Academy of Sciences of the United States of America* **118** (2021).

13. Anderson, R.L. et al. Deciphering the super relaxed state of human beta-cardiac myosin and the mode of action of mavacamten from myosin molecules to muscle fibers. *Proc. Natl. Acad. Sci. U. S. A.* **115**, E8143-E8152 (2018).

14. Winkelmann, D.A., Forgacs, E., Miller, M.T. & Stock, A.M. Structural basis for drug-induced allosteric changes to human beta-cardiac myosin motor activity. *Nature communications* **6** (2015).

15. Usaj, M., Moretto, L., Vemula, V., Salhotra, A. & Mansson, A. Single molecule turnover of fluorescent ATP by myosin and actomyosin unveil elusive enzymatic mechanisms. *Commun Biol* **4**, 64 (2021).

16. Vemula, V., Huber, T., Usaj, M., Bugyi, B. & Mansson, A. Myosin and gelsolin cooperate in actin filament severing and actomyosin motor activity. *J. Biol. Chem.* **296**, 100181 (2021).

17. Moretto, L. et al. Multistep orthophosphate release tunes actomyosin energy transduction. *Nature communications* **13**, 4575 (2022).
